# Knowledge, attitudes, practices, and its associated factors toward COVID-19 pandemic among Bangladeshi older adults

**DOI:** 10.1371/journal.pone.0275065

**Published:** 2022-12-15

**Authors:** Deepa Roy, Suvasish Das Shuvo, Md. Sakhawot Hossain, Md. Riazuddin, Sanaullah Mazumdar, Bappa Kumar Mondal, Md. Ashrafuzzaman Zahid

**Affiliations:** 1 Department of Mathematics, Jashore University of Science and Technology, Jashore, Bangladesh; 2 Department of Nutrition and Food Technology, Jashore University of Science and Technology, Jashore, Bangladesh; 3 Nutrition International, Dhaka, Bangladesh; Unaizah College of Pharmacy, Qassim University, SAUDI ARABIA

## Abstract

**Background:**

The newly emerged COVID-19 has an unprecedented impact on all classes of people, particularly the elderly. The knowledge, attitudes, and practices (KAP) of older adults toward COVID-19 are currently unknown. This study aimed to investigate the KAP and its associated factors toward COVID-19 among older adults in Bangladesh.

**Methods:**

A cross-sectional survey was conducted from April to May 2021 among Bangladeshi older adults. Face-to-face interviews were used to collect data from five selected divisions in Bangladesh using simple random sampling. The questionnaire consisted of socio-demographic characteristics, disease conditions, and KAP toward COVID-19. Descriptive statistics, t-tests, one-way analysis of variance (ANOVA), and logistic regression analyses were performed.

**Results:**

Out of 900 respondents, the majority of older adults (82.9%) indicated that COVID-19 is a viral disease and the major clinical symptom of COVID-19 (86.5%). Only 22.1% of participants always washed their hands with soap or hand sanitizer, and 27.6% wore a mask to protect against the virus when going outside the home. Overall, 55.2% had adequate knowledge, 50.2% had positive attitudes toward COVID-19 and only 22.7% had good practices. Out of 30 scores, mean score values were 20.8±6.7 in the knowledge section, 21.2±4.3 in the attitude section, and 11.3±6.7 in the practice section out of 30. In binary logistic regression analysis, factors associated with poor knowledge, and practices were being male, aged >70 years, having a primary education, less income <5000BDT, and multimorbidity (p < 0.05). Participants having poor knowledge of COVID-19 had higher likelihood of negative attitudes (OR: 6.79, 95% CI = 4.87–9.47, p < 0.001) and poor practices (OR: 9.15, 95% CI = 6.94–13.16, p < 0.001).

**Conclusion:**

The findings highlight the need for immediate implementation of health education programs and adequate intervention programs for COVID-19 which integrates consideration of associated factors to improve the level of older adults’ knowledge, attitudes, and practices.

## Introduction

The first confirmed case of Severe Acute Respiratory Syndrome Coronavirus 2 (SARS-CoV-2) or COVID-19 was reported in late 2019 in Wuhan, China. Since then, the situation has gotten worse, and the World Health Organization declared it a public health emergency of international concern (PHEIC) on January 30, 2020. (WHO) [1]. Being a contagious disease, the virus rapidly spread worldwide, and observing its severity, WHO declared the outbreak a global pandemic on 11 March 2020 [2]. To date (11^th^ May 2022), the total number of confirmed cases is 518,566,678 with more than 6,280,390 deaths across the globe [3]. The Institute of Epidemiology, Disease Control and Research (IEDCR) in Bangladesh announced the first-ever confirmed case of COVID-19 on March 8, 2020, and the number of cases has continued to rise since then, with a high mortality rate [4, 5]. Since the Vaccines for COVID-19 were not very much available for all classes of people, the Government had to take non-therapeutic measures including lockdown, self-isolation or quarantine, and social distancing which was started on 26 March 2020 by the declaration of a nation wise lockdown. As the country has not experienced any other epidemics such as SARS or MERS, the public health care systems were not prepared for this pandemic although the Government has taken various measures to upgrade the healthcare system to fight against COVID-19 [6, 7], still, this is not up to the mark to slow down the rapid spread and at the same time to stop the increasing mortality rate. As of 11 May 2022, the total number of confirmed cases was 1,952,855 where 1,898,063 were recovered but at the same time, the country has recorded 29127 death cases [8, 9].

As an infectious disease, the virus spreads mostly through intimate contact, and infected people experience a wide range of symptoms, including fever, cough, shortness of breath, loss of smell, dyspnea, headache, sore throat, and rhinorrhea [10]. According to research, the transmission dynamics of COVID-19 are based on two mechanisms: mechanisms: human-to-human transmission (measured by population density) and air pollution to humans (measured by airborne viral infectivity). COVID-19 poses a threat to people of all ages, but the elderly and those with pre-existing medical disorders are particularly susceptible to serious infections and even death as a result of the virus [11–13]. Clinical evidence suggests that the disease is more severe in older people or those with underlying diseases, such as heart disease, hypertension, diabetes, lung, and kidney disease, which weaken their body’s ability to fight infectious diseases [10, 11, 14]. The risk is magnified for older people in such an environment by not only the poor living environment but also their advanced age that compromises their immune system, the presence of underlying chronic illnesses [15], and cognitive impairment, which causes difficulty in their participating in proper prevention activities [16].

In Bangladesh, among the reported deaths the percentage of the age group 65–74 is 30.5 followed by the group of 55–64 (12%) [17]. In addition, the death rate was higher among men who were 70 years old and had a history of heart problems, high blood pressure, kidney disease, or diabetes [5]. In point of fact, in the early stages of the COVID-19 outbreak, older individuals underestimated the severity of the epidemic because it only impacted a relatively small population. They were reluctant to listen to the authorities, believing that there was no need to practice the suggested preventive behaviors for COVID-19. A recent study found that compared with younger adults, older men were less worried about COVID-19 and adopted fewer preventive behaviors [18]. It is evident that any misunderstanding or misconception about emerging infectious diseases can easily turn into anxiety, disorder, and even extreme fear that may worsen the current pandemic situation [19]. Hence, special efforts should be made to enhance older people’s protection against this new infectious disease. A good understanding of underlying factors that encourage older people to adopt particular preventive behaviors is significant to providing necessary strategies to implement. A higher level of knowledge has been proven to be associated with positive behavior changes [19]. In addition, studies on individuals’ behavior changes toward infectious diseases have suggested that perceptions or beliefs about an outbreak are important in determining the adoption of particular preventive behaviors [20].

Several studies had also been conducted on COVID-19-related knowledge, attitudes, and practices among older people in different countries including Thailand [21], Kenya [22], the U.S.A [23], China [24], and Iran [25]. In Bangladesh, studies have investigated behavioral responses toward COVID-19 among the general population [26–28] and young adults [29]. Despite the detection of increased vulnerability among this population in Bangladesh, literature reports solely focusing on behavior changes in older people remain scant, especially in multimorbid patients. To prevent this pandemic from having serious implications on such highly vulnerable groups, it is imperative to institute effective infection prevention and control measures. Consequently, it is urgent to understand their knowledge, perception, assessment of the risk, and adherence to prevention measures [23]. Therefore, this study aimed to assess the knowledge, attitudes, practices, and its associated factors regarding the COVID-19 pandemic among older Bangladeshi residents. Our findings may help to enhance the effectiveness of intervention programs targeting the older population and also help to prepare possible steps amid further waves.

## Methods

### Study design and data collection

A community-based cross-sectional study was conducted to evaluate the knowledge, attitudes, practices, and its associated factors regarding the COVID-19 pandemic among older adults during the second phase of the COVID-19 pandemic from early April to late May 2021. This study was conducted using multistage sampling. In this study, five divisions of Bangladesh (Dhaka, Chattogram, Rajshahi, Mymensingh, and Khulna) were selected using convenience sampling methods because of movement restrictions caused by the countrywide strict lockdown. Then five urban and five rural areas from each division were randomly included for data collection. We estimated the sample size based on an unknown prevalence of household food security (therefore considering 50% prevalence) with a 5% margin of error to be tolerated at the 95% level of confidence, and 95% response rate. Primarily, 965 potential older adult participants were approached, and only 900 eligible participants agreed to participate. An initial draft of the questionnaire was validated by an expert reviewing it. A pilot study was conducted among 50 respondents to confirm the reliability of the questionnaire before the inauguration of the final data collection. The inclusion criteria to participate in the study were being a Bangladeshi resident, aged ≥55 years old and above agreed to participate in the study using a simple random sampling technique. Respondents who were being <55 years old, did not consent to the survey willingly, and were intellectually disabled or unable to communicate were excluded from the survey. The questionnaire was drafted in English and then translated to Bengali. It was retranslated back to English to ensure the meaning of the content and then verified through back-translation. All respondents were clearly informed about the study’s objective and were free to withdraw at any time, without giving a reason. All information and opinions provided would be anonymous and confidential. Respondents who agreed to participate in the study were asked to sign a consent form.

### Measures

A structured questionnaire was designed by referring to the available information from the World Health Organization, the Center for Disease Control and Prevention (USA), and the Ministry of Health and Family Welfare Bangladesh, and guidelines suggested by the country’s Institute of Epidemiology, Disease Control and Research (IEDCR) [30–32]. The questionnaire consisted of four sections including socio-demographic characteristics, knowledge, attitudes, and practices associated with COVID-19.

### Outcome measures

Knowledge, attitudes, and practices were the primary outcome measured using the 45 questions regarding COVID-19 [26, 28]. The knowledge part consisted of 15 items regarding the general knowledge of respondents about the possible spread, transmission, signs and symptoms, risk factors, and prevention of COVID-19 with three possible answers. The answer included yes was coded as 2, not sure was coded as 1, and no was coded as 0. The total score range was 0–30.

The attitude section comprised fifteen questions that assessed the behavioral perception of prevention concerning COVID-19 and the response of each question indicated their level of agreement to the statements using three points rating scale strongly agree (coded as 2), agree (coded as 1), and disagree (coded as 0). The score range was calculated by summating the raw scores of the fifteen questions ranging from 0 to 30. Lastly, the practice section includes 15 questions about practices towards the COVID-19 pandemic with three possible answers coded as 2 for always, 1 for sometimes, and 0 for never. The maximum and minimum scores were 0 and 30. A cut-off score level of ≥18 was set for adequate knowledge, positive attitudes, and good practices whereas a score level of ≤18 was set for poor knowledge, negative attitudes, and poor practices, respectively [26, 33].

### Explanatory variables

Explanatory variables considered in this study were gender (male or female), age (categorized as 55–62 years, 63–70 years, and >70 years), education level (higher secondary and above, secondary school, and primary school), marital status (married or widowed), family size (≤4 or >4), household income per month (<5000 BDT, 5,000–10,000 BDT, and >10,000 BDT), residence (rural or urban), living arrangements (living alone or with family), disease condition (no condition, only one condition, and multi-morbidity). Self-reported data on pre-existing disease conditions, such as osteoarthritis, diabetes, hypertension, heart disease, chronic respiratory disease, stroke, hypercholesterolemia, chronic kidney disease, and cancer were collected. This information was double-checked with medical records (if available and/or family members).

### Data analysis

Data entry and analysis were performed by using STATA version 14.0. Data editing, sorting, and coding was used by using STATA software. The normality of distribution of knowledge, attitude, and practice variables was checked by the Kolmogorov-Smirnov (K-S) test and for homogeneity of variances using Levene’s test. Data analysis consisted of descriptive statistics, including frequency distribution, percentage, and computing mean scores. In addition, t-tests or one-way ANOVA tests were performed to determine significant relations of the mean KAP scores with the sociodemographic variables of the participants. Binary logistic regression was performed with a 95% confidence interval (95% CI) to determine the significant associations between categorical dependent and independent variables and *p*-value ≤0.05 were considered significant.

### Ethical considerations

This study’s protocol was approved by the Research Ethical Committee (REC) of the Faculty of Biological Science and Technology, Jashore University of Science and Technology, Jashore, 7408, Bangladesh. The Institutional ethics review board of Jashore University of Science and Technology approved formal ethics approval (Ref. No.: ERC/FBST/JUST/2021-59). The procedure and purpose of the study were explained to the participants, and anonymity and confidentiality were strictly maintained. During the interview periods, all potential participants willingly consented to be a part of the study and signed a consent form.

## Results

Socio-demographic characteristics and disease conditions of the studied respondents have shown in Table 1. Nearly two-thirds (63.7%) were males. The majority of the study respondents were aged between 63–70 years (40.5%) and 63–70 years (42.7%). More than 42% of respondents had secondary level of education. Among the participants, more than three fourth (77.1%) of the participants were married and 62.4% had >4 people in their family at the time of the study. The monthly income of a large proportion of the participants (42.3%) ranged from 5,000–10,000 BDT. Besides, 54.4% were urban residents, 71.4% of unemployed, and 83.9% of the household lived with family, respectively. More than one-third (38.1%) of the participants had only one clinical condition and 29.1% of all participants had multiple clinical conditions (>2 clinical conditions).

**Table 1:** Socio-demographic and clinical characteristics of the older people (n = 900).

Variables	Frequency (%)
**Gender**	
Female	327 (36.3)
Male	573 (63.7)
**Age**	
55–62 years	365 (40.5)
63–70 years	384 (42.7)
>70 years	151 (16.8)
**Education level**	
Higher secondary and above	291 (32.3)
Secondary School	384 (42.7)
Primary School	225 (25.0)
**Marital status**	
Widowed	206 (22.9)
Married	694 (77.1)
**Family size**	
<4	338 (37.6)
>4	562 (62.4)
**Household income per month**	
<5000 BDT	358 (39.8)
5,000–10,000 BDT	381 (42.3)
>10,000 BDT	161 (17.9)
**Residence**	
Rural	410 (45.6)
Urban	490 (54.4)
**Occupation**	
Unemployed	643 (71.4)
Employed	257 (28.6)
**Living arrangement**	
Live alone	145 (16.1)
Live with family	755 (83.9)
**Disease condition**	
No condition	295 (32.8)
Only one condition	343 (38.1)
Multi-morbidity	262 (29.1)

*BDT = Bangladeshi Taka

### Assessment of knowledge

A total of fifteen questions were used to measure knowledge regarding the COVID-19 pandemic (Table 2). The majority of the respondents reported that COVID-19 is a viral disease (83%) and fever, fatigue, dry cough, and myalgia are the major clinical symptoms of COVID-19 (86%). Additionally, 71.2% correctly reposed the importance of 14 days period in case of home quarantine, and 76.4% of respondents were also well aware of the availability of a vaccine against Covid-19 in your area. Although 67.3% of the respondents agreed that COVID-19 is an airborne virus, 69.1% positively answered that the duration of hand washing is necessary for preventing COVID-19. However, 33.8%, 33.9%, and 35.2% negatively answered questions regarding social distancing, wearing face masks can prevent infection with COVID-19, and the mode of transmission of Covid-19.

**Table 2:** Respondents’ knowledge towards COVID-19 pandemic (n = 900).

	Frequency (%)		
Question	Yes	No	Don’t know
1. Is COVID-19 a viral disease?	746 (82.9)	65 (7.2)	89 (9.9)
2. Do you know the major clinical symptoms of COVID-19 are fever, fatigue, dry cough, and myalgia?	778 (86.5)	57 (6.3)	65 (7.2)
3. Are People infected with COVID-19 cannot transmit the virus to others when a fever is not present?	408 (45.3)	208 (23.1)	284 (31.6)
4. Do you know COVID-19 can spread by infected hands touching the face, mouth and eyes?	577 (64.1)	193 (21.4)	130 (14.5)
5. Do you know the virus of COVID-19 is airborne?	606 (67.3)	133 (14.8)	161 (17.9)
6. Is any importance of 14 days period in case of home quarantine?	641 (71.2)	56 (6.2)	203 (22.6)
7. Do you know the availability of vaccine against Covid-19 in your area?	688 (76.4)	116 (12.9)	96 (10.7)
8. Do know what is social distancing?	492 (54.7)	304 (33.8)	104 (11.5)
9. Do you know Ordinary residents can wear face masks to prevent infection with COVID-19?	439 (48.8)	305 (33.9)	156 (17.3)
10. Do you know the mode of transmission of Covid-19?	433 (48.1)	317 (35.2)	150 (16.7)
11. Do you know COVID-19 patients develop severe acute respiratory illness?	478 (53.1)	257 (28.6)	165 (18.3)
12. Do you know the duration of hand washing necessary for preventing COVID-19?	622 (69.1)	146 (16.2)	132 (14.7)
13. Do you know that to prevent infection by COVID-19, individuals should avoid going to crowded places and taking public transport?	442 (49.1)	227 (25.2)	231 (25.7)
14. Do you know people with comorbidity like diabetes, hypertension, heart disease etc. are at risk of developing COVID-19?	493 (54.8)	288 (32.0)	119 (13.2)
15. Do you know Isolation and treatment of people who are infected with the COVID-19 virus are effective ways to reduce the spread of the virus?	393 (43.7)	354 (39.3)	153 (17.0)
		**Adequate**	**Poor**
**Total Knowledge level**		497 (55.2)	403 (44.8)

The association between socio-demographic characteristics and knowledge about COVID-19 is shown in Table 5. The knowledge score was significantly lower in males (19.4± 7.2) (p = 0.010). There were statistically significant differences between knowledge mean scores of different age groups (p < 0.001), where significantly lower knowledge mean scores were obtained for respondents aged >70 years and those 55–62 years (20.98±6.6 and 19.3±7.7 respectively) compared to 63–70 years age groups. Also, the rural areas residents had a significantly lower mean score (20.2±6.93) compared to urban areas residents (21.5 ±6.4); (p<0.001). The respondent’s knowledge score was significantly associated with the household-heads’ occupation. The knowledge mean scores were significantly related to the level of education as well as to the monthly income (p < 0.001). Participants with primary education had significantly lower knowledge mean scores compared to those with secondary and higher levels of education. Similarly, participants with monthly incomes of less than 5000 BDT had significantly lower knowledge mean scores compared to participants with higher incomes. Surprisingly, the knowledge score was significantly lower in multimorbid respondents as compared no condition peers.

### Assessment of attitudes

Majority respondents (83%) had positive attitudes about the COVID-19 pandemic (Table 3). About 94% of the participants agreed that washing hands and face is important after coming outsides. Besides, 81.2%, 91.6%, and 75.3% of respondents had positive attitudes regarding the use of a face mask is important in a crowded place, lockdown can play a vital role to minimize COVID-19 patients, and keep distance from others is important to avoid spreading COVID- 19, respectively. Morover, 83.7% and 76.9% of respondents agreed that COVID-19 patients should be kept in isolation in health facilities and acceptance of isolation in health facilities if getting COVID-19. Remarkably, 68.9%, 37.4%, and 56.8% of respondents did not have any idea regarding antibiotics as treatment of choice for COVID- 19, money can be a mode of transmission for COVID-19, and nutritious food is enough to fight against COVID-19, respectively (Table 3). However, a statistically significant association between attitude and socio-demographic variables such as age groups, education levels, occupation, and living arrangement (p< 0.001). Table 5 explains that the participants’ attitudes score was significantly higher among 63–70 years of age (21.9±4.3, *p<*0.001), and secondary level of education (22.6 ± 4.2, *p<*0.001). Again, unemployed (20.5 ± 3.9, p<0.001) and living alone participants (20.3± 3.97, p<0.001) had significantly lower attitudes scores than others.

**Table 3:** Respondents attitude towards COVID-19 pandemic (n = 900).

	Frequency (%)		
Question	Agree n (%)	Disagree n (%)	No idea n (%)
1. Do you think it is important to wash hands and face after coming outsides?	844 (93.8)	16 (1.8)	40 (4.4)
2. Is it important to keep a distance from others, to avoid spreading COVID- 19?	739 (82.1)	89 (9.9)	72 (8.0)
3. Do you think COVID-19 cases can be treated at home?	334 (37.1)	141 (15.7)	425 (47.2)
4. Do you think antibiotics are a treatment of choice for COVID- 19?	81 (9.0)	199 (22.1)	620 (68.9)
5. Do you think COVID-19 will eventually be successfully controlled?	153 (17.0)	327 (36.3)	420 (46.7)
6. Is Bangladesh in a good position to control COVID-19?	148 (16.5)	676 (75.1)	76 (8.4)
7. Is it important to use a face mask in a crowded place?	824 (91.6)	28 (3.1)	48 (5.3)
8. Do you think lockdown can play a vital role to minimize COVID-19 patients?	678 (75.3)	154 (17.1)	68 (7.6)
9. Do you think COVID-19 patients should be kept in isolation in health facilities?	753 (83.7)	23(2.5)	124(13.8)
10. Will you accept isolation in health facilities if getting COVID-19?	692 (76.9)	121 (13.4)	87 (9.7)
11. Do you think COVID-19 can be prevented by avoiding public transportation?	509 (56.6)	135 (15.0)	256 (28.4)
12. Can health education play an important role in COVID-19 prevention?	598 (66.4)	42 (4.7)	260 (28.9)
13. Do you think money can be a mode of transmission for COVID-19?	507 (56.4)	56 (6.2)	337 (37.4)
14. Do you think washing fruits and vegetables brought from the market can reduce the rate of COVID-19 patients?	421 (46.8)	39 (4.3)	440 (48.9)
15. Do you think nutritious food is enough to fight against COVID-19?	356 (39.6)	33 (3.7)	511 (56.8)
		**Positive**	**Negative**
**Total Attitudes level**		452 (50.2)	448 (49.8)

### Assessment of practices

In terms of practices toward COVID-19, participants found that 38.2% and 49.3% never washed their hands using soaps or hand sanitizer frequently and never avoided cultural behaviors, such as shaking hands (Table 4). In particular, 63.1% of respondents never washed their hands or use alcohol-based sanitizer after touching or shaking hands with others. On the other hand, 65.2% and 58.1% of respondents reported that passersby did not use a mask when they talked with them and did not maintain a healthy lifestyle focusing on the COVID-19 outbreak, respectively. In addition, 54.6% and 56.4% never ate healthy food focusing on the outbreak, and never avoided touching their eyes, nose, and mouth with unwashed hands.

**Table 4:** Respondents practice toward COVID-19 pandemic (n = 900).

	Frequency (%)		
Question	Never	Occasionally	Always
1. Do you wash your hands frequently using soap or hand sanitizer?	344 (38.2)	357 (39.7)	199 (22.1)
2. Have you recently avoided cultural behaviors, such as shaking hands?	444 (49.3)	237 (26.3)	219 (24.4)
3. After touching or shaking hands with others do you wash your hands or use alcohol-based sanitizer?	568 (63.1)	155 (17.2)	177 (19.7)
4. While coughing and sneezing, Do you cover your mouth and nose with an elbow or tissue?	324 (36.0)	408 (45.3)	168 (18.7)
5. When you talked with others, are they use the mask?	587 (65.2)	194 (21.6)	119 (13.2)
6. Do you maintain a healthy lifestyle focusing on the outbreak?	523 (58.1)	256 (28.4)	121 (13.5)
7. Do you spit in the public area?	361 (40.1)	306 (34.0)	233 (25.9)
8. Do you wear a mask to protect against the virus?	262 (29.1)	390 (43.3)	248 (27.6)
9. Recently, have you frequently washed your hands with soap and water, for at least 40 seconds, especially after going to a public place, or after nose-blowing, coughing, or sneezing?	466 (51.8)	208 (23.1)	226 (25.1)
10. Have you recently been to a social event involving a large number of people?	280 (31.1)	296 (32.9)	324 (36.0)
11. Are you practicing social distancing of at least 1 meter (3 feet)?	464 (51.6)	256 (28.4)	180 (20.0)
12. Do you dispose of your mask after using it?	534 (59.3)	218 (24.2)	148 (16.5)
13. Do you eat healthy food focusing on the outbreak?	491 (54.6)	205 (22.8)	204 (22.6)
14. Do you avoid touching your eyes, nose, and mouth with unwashed hands?	508 (56.4)	252 (28.0)	140 (15.6)
15. Do you avoid sick (they have a fever, cough, or other symptoms) people?	460 (51.1)	226 (25.1)	224 (23.8)
		**Good**	**Poor**
**Total Practices level**		204 (22.7)	696 (77.3)

Table 5 shows that participants’ mean practice score was significantly different in terms of gender, age groups, education level, residence, occupation, and disease condition (p < 0.05). The participants’ practice score was significantly higher among females versus males (11.5±6.6, *p <* 0.05) and 63–70 years of age (12.1±6.8, p<0.001). The result also shows that the practice score was significantly higher in secondary education (13.6±7.2, p<0.001) compared to others. The mean knowledge score was found to be 10.8 (±6.7) and 9.9 (±5.99) with living in rural areas and unemployment having a significantly lower mean of knowledge (P< 0.05). Moreover, their mean practices score was significantly lower in multimorbid respondents (p<0.05).

**Table 5:** Association between sociodemographic characteristics and mean KAP score among Bangladeshi older people (n = 900).

Variables	Knowledge		Attitude		Practice	
	Mean (SD)	F/t-test	Mean (SD)	F/t-test	Mean (SD)	F/t-test
**Gender**						
Male	19.4 (7.2)	25.21***	20.9 (3.9)	1.38	10.7 (6.8)	3.32**
Female	21.6 (6.3)		21.3 (4.4)		11.5 (6.6)	
**Age**						
55–62 years	20.98 (6.6)	4.46***	20.8 (4.09)	9.67***	10.6 (6.2)	7.44***
63–70 years	21.2 (6.4)		21.9 (4.3)		12.1 (6.8)	
>70 years	19.3 (7.7)		20.2 (4.5)		10.1 (7.0)	
**Education**						
Higher secondary and above	20.04 (6.8)	24.63***	20.3 (3.7)	43.40***	9.4 (5.9)	45.46***
Secondary School	22.5 (6.6)		22.6 (4.2)		13.6 (7.2)	
Primary School	19.1 (6.1)		19.8 (4.2)		9.5 (5.3)	
**Marital status**						
Widowed	19.8 (6.8)	6.08	21.3 (4.1)	0.14	11.1 (6.98)	0.11
Married	21.1 (6.7)		21.1 (4.3)		11.3 (6.6)	
**Family size**						
<4	20.1 (6.3)	4.3***	21.1 (3.88)	0.08	10.7 (5.8)	1.86
>4	21.1 (6.96)		21.2 (4.5)		11.5 (7.1)	
**Family income**						
<5000 BDT	19.4 (6.91)	2.19***	19.1 (4.5)	1.46	10.6 (6.0)	2.10
5,000–10000 BDT	21.3 (6.5)		20.97 (4.1)		11.2 (6.6)	
>10,000 BDT	20.4 (6.7)		21.7 (4.1)		10.3 (5.87)	
**Residence**						
Urban	21.5 (6.4)	7.68***	21.5 (4.2)	4.10**	11.8 (6.6)	4.87**
Rural	20.2 (6.93)		20.9 (4.3)		10.8 (6.7)	
**Occupation**						
Unemployed	19.7 (6.6)	44.86***	20.5 (3.9)	49.77***	9.9 (5.99)	94.78***
Employed	23.1 (6.3)		22.7 (4.6)		14.5 (7.1)	
**Living arrangement**						
Live alone	20.6 (8.06)	0.13	20.3 (3.97)	6.91***	10.7 (6.7)	0.86
Live with family	20.8 (6.4)		21.3 (4.3)		11.3 (6.7)	
**Disease condition**						
No condition	21.2 (6.6)	5.43***	21.5 (4.6)	1.20	11.5 (6.5)	1.48**
Only one Condition	21.2 (7.01)		20.99 (4.6)		11.4 (6.9)	
Multimorbidity	19.6 (6.4)		21.1 (3.4)		10.6 (6.6)	
**Total mean score**	**20.8 (6.7)**		**21.2 (4.3)**		**11.3 (6.7)**	

SD = Standard deviation

*p < 0.05

**p < 0.01

***p < 0.001

### Factors associated with poor knowledge, negative attitudes, and poor practices toward COVID-19

Regression analysis revealed factors associated with poor knowledge of the participants and found that male participants had higher odds of having poor knowledge (vs. female, OR: 1.56, 95% CI: 0.62–2.55, p < 0.001). Similarly, participants who had >70 years old (vs. 55–62 years old OR: 1.08, 95% CI: 0.71–1.61, p < 0.05), education of “primary school” (vs. higher secondary and above, OR: 1.88, 95% CI: 1.03–2.02, p < 0.001), monthly family income below 5000BDT (vs. above 10000 BDT, OR: 2.85, 95% CI: 0.56–1.2, p < 0.001), lived in a rural area (vs. urban, OR: 1.47, 95% CI: 1.11–1.96, p < 0.001), and multimorbidity (vs. no condition, OR: 1.38, 95% CI: 0.96–1.98, p < 0.05) had higher odds of having poor knowledge regarding COVID-19 (Table 6).

**Table 6:** Factors associated with poor knowledge, negative attitudes, and poor practices toward COVID-19 in using a multivariate logistic regression model (n = 900).

Variables	Poor knowledge	Negative attitude	Poor practice
	*OR (95% CI)*	*OR (95% CI)*	*OR (95% CI)*
**Gender**			
Female	Reference	Reference	Reference
Male	1.56 (0.62–2.55)***	1.07 (0.80–1.45)	2.10 (0.75–3.48)**
**Age**			
55–62 years	Reference	Reference	Reference
63–70 years	1.05 (0.77–1.44)	0.72 (0.52–0.98)**	0.63 (0.42–0.94)
>70 years	1.08 (0.71–1.61)**	0.91 (0.60–1.38)	1.68 (0.89–2.55)***
**Education**			
Higher secondary and above	Reference	Reference	Reference
Secondary School	0.45 (0.32–0.65)	0.30 (0.21–0.43)***	0.27 (0.17–0.43)**
Primary School	1.88 (0.60–1.2)***	0.94 (0.64–1.38)	2.62 (1.34–3.94)***
**Marital status**			
Widowed	Reference	Reference	Reference
Married	0.92 (0.66–1.3)	1.03 (0.73–1.46)	1.43 (0.92–2.23)*
**Family Size**			
<4	Reference	Reference	Reference
>4	0.63 (0.47–0.85)***	1.04 (0.77–1.39)	1.43 (0.29–1.55)***
**Family Income**			
>10,000 BDT	Reference	Reference	Reference
5,000–10,000 BDT	0.54 (0.36–0.81)	1.86 (1.23–2.80)**	0.64 (0.36–1.14)
<5000 BDT	2.85 (0.56–1.2)***	2.02 (1.32–3.10)***	2.63 (0.36–1.12)***
**Residence**			
Urban	Reference	Reference	Reference
Rural	1.47 (1.11–1.96) ***	1.33 (0.99–1.77)**	1.36 (0.94–1.97)**
**Occupation**			
Unemployed	Reference	Reference	Reference
Employed	0.55 (0.39–0.77)***	0.43 (0.30–0.60)***	0.20 (0.14–0.30)***
**Living arrangement**			
Live alone	Reference	Reference	Reference
Live with family	1.31 (0.87–1.98)	0.70 (0.47–1.06)**	0.32 (0.17–0.60)***
**Disease condition**			
No condition	Reference	Reference	Reference
Only one condition	1.03 (0.73–1.46)	1.13 (0.79–1.61)	1.06 (0.65–1.71)
Multimorbidity	1.38 (0.96–1.98)**	1.02 (0.70–1.47)	1.33 (0.85–2.07)**
**Knowledge level**			
Adequate		Reference	Reference
Poor		6.79 (4.87–9.47)***	9.15 (6.94–13.16)***
**Attitude level**			
Positive			Reference
Negative			9.81 (5.97–16.12)***

OR = Odds ratio, CI = Confidence interval

*p < 0.05

**p < 0.01

***p < 0.001

Participant with monthly family income below 5000BDT (vs. above 10000 BDT, OR: 2.02, 95% CI: 1.32–3.10, p < 0.001), lived in a rural area (vs. urban, OR: 1.33, 95% CI: 0.99–1.77, p < 0.05), and having poor knowledge (vs. adequate, OR: 6.79, 95% CI: 4.87–9.47, p < 0.001) were more likely to have a positive attitude (Table 6). In addition, respondents age group in 63–70 years (vs. 55–62 years old OR: 0.72, 95% CI: 0.52–0.98, p < 0.05), education of “secondary school” (vs. higher secondary and above, OR: 0.30, 95% CI: 0.21–0.43, p < 0.001), employed respondents (vs. unemployed, OR: 0.43, 95% CI: 0.30–0.60, p < 0.001), and respondents lived with family (vs. lived alone, OR: 0.70, 95% CI: 0.47–1.06, p < 0.05) were less likely to have a negative attitude (Table 6).

Regression analysis showed that male gender (vs. female, OR: 2.10, 95% CI: 0.75–3.48, p < 0.05), education of “primary school” (vs. higher secondary and above, OR: 1.88, 95% CI: 1.03–2.02, p < 0.05), respondents age group in above 70 years (vs. 55–62 years old OR: 1.68, 95% CI: 0.89–2.55, p< 0.001), education of “primary school” (vs. higher secondary and above, OR: 2.62, 95% CI: 1.34–3.94, p < 0.001), family size >4 (vs. family size <4, OR: 1.43, 95% CI: 0.29–1.55, p < 0.001), monthly family income below 5000BDT (vs. above 10000 BDT, OR: 2.63, 95% CI: 0.36–1.12, p < 0.001), lived in a rural area (vs. urban, OR: 1.36, 95% CI: 0.94–1.97, p < 0.05), multimorbidity (vs. no condition, OR: 1.33, 95% CI: 0.85–2.07, p < 0.05), negative attitude (vs. positive attitude, OR: 9.15, 95% CI: 6.94–13.16, p < 0.001), and having poor COVID-19 knowledge (vs. adequate, OR: 9.81, 95% CI: 5.97–16.12, p < 0.001) were more likely to have poor practices (Table 6).

## Discussion

From the beginning of COVID-19 outbreaks, we have seen that older people are more vulnerable [11, 14] than younger in sense of severity and death rate. Older people are most susceptible to being affected by different acute diseases and now the COVID-19 outbreaks are severely affecting their health [11]. Therefore, considering the previous study; knowledge about COVID-19 infection is very much important to fight against it [24]. To prevent the spread of COVID-19 among older people, the adoption of health-related behavior might be the first step. So implementation of unique strategy should have to implement to encourage older people to practice precautionary behaviors.

In this study, it was found that Bangladeshi older adults had good knowledge of COVID-19; the respondents had an overall correct score of 20.8 on the knowledge questionnaire, which was lower than the rate reported for the Chinese older population (90%) [24, 25] but much higher than that reported for Kenya, and Thailand older residents (43%) [21, 22]. This finding may be due to the large amounts of publicity related to COVID-19 through various channels that are appropriate to the needs and characteristics of older people, such as vivid prints, marked banners, and broadcasts in dialect. The study also showed that the mean score of knowledge was significantly related to gender, age, education, family size, family income, residence, and disease conditions in older adults. Whereas, another previous study agreed that poor knowledge was suggestively related to different demographic and socioeconomic factors of individuals [34]. From regression analysis, it was found that poor knowledge was associated with gender, age, education, family size, family income, residence, and disease conditions. Lower education level was significantly associated with having poor knowledge among Bangladeshi older people, which was also mentioned in other studies [35, 36]. This might be the cause of limitations to watch, read and understand the available and recommended information. Similarly, cognitive status and lack of familiarity with modern technologies (social networks/ internet) might be another reason for poor knowledge among older adults [37]. This study also found that lower family income negatively influenced older adults’ knowledge, attitude, and practice. The findings of this study are partially consistent with those of another research project conducted in Bangladesh, which found that farmers and daily laborers were more likely to have low levels of knowledge [34]. More than one-third of our study participants had lower family incomes, who were severely affected during the nationwide extended lockdown. They immediately lost their job as well as their source of income, which may limit their access to reliable sources of information on COVID-19.

As with the good knowledge score, the findings also reported that older people showed a- positive attitude toward COVID-19. Older people showed a very positive attitude regarding the issue of wearing face masks before going to crowded places (91.6%) following other recommendations. These findings were quite similar to a study conducted at the beginning of COVID-19 outbreaks in Bangladesh (98.7%) [26] and in China (98%), during the rapid rise of the COVID-19 outbreak [36]. This positive attitude is also found among most health care professionals towards wearing protective gear [38]. Similarly, most of the respondents agreed that wash hands and face after coming from outside (93.8%) and keeping a distance from others (82.1%) can play an important role in COVID-19 prevention. Like this study, Chen et al. found similar attitudes among Chinese older adults [24]. Although many older people did not have any idea regarding antibiotics being a treatment of choice for COVID- 19, money can be a mode of transmission for COVID-19, and nutritious food is enough to fight against COVID-19. As in other studies [14, 36], this study also found that more than three-fourth percent of respondents agreed that lockdown can play a vital role to minimize COVID-19, and COVID-19 patients should be kept in isolation in health facilities. Surprisingly, there is a significant association between attitude and socio-demographic variables (age, education, occupation and living arrangement), a similar result was found in an Iranian study that mentioned attitude score had significant relation with age but no relation with sex and history of underlying diseases [25]. Alternatively, other studies found participants’ attitudes were not affected by age, gender, experience, and occupation [39] but found a statistically significant association with occupation [40]. Similar to other studies, this study also identified that participants’ socio-demographic status had a significant relationship with their safety behaviors [24]. The regression analysis showed that the participants with higher family income, higher education, adequate knowledge, full-time employment, and family living arrangements were less likely to have a negative attitude, a finding consistent with other prior research on COVID-19 [25, 26] and also regarding SARS [41]. On the other hand, we did not find any significant relationship between participants’ attitudes with age, which was similar to other study [24]; meanwhile, another study reported that younger participants were implementing more preventive measures compared with older (>60 years) participants [18].

In terms of practices, the study identified a lower mean practice score among older adults in Bangladesh. Considering the key practice recommendations for COVID-19; we found that 22.1% of participants always washed their hands using water and soaps frequently, which was lower than Kenyan older adults (69.8%) [22] and Bangladeshi general people (93.8%) [26]. Moreover, 63.1% of respondents never washed their hands or use alcohol-based sanitizer after touching or shaking hands with others, and 65.2% of respondents reported that passersby did not use masks when they talked with them, which is consistent with a study conducted by Daoust JF [14]. Despite their increased susceptibility, the elderly are not disciplined enough to adopt the necessary COVID-19 safety measures. This poor practice may be a cause of the rapid spread of infectious diseases like COVID-19 and Bangladesh has never faced any pandemic situation like this [34]. To reduce societal unrest, many countries have imposed lockdown policies ranging from a single city to the rest of the country [21, 24, 25, 35]. Alternatively, the Bangladeshi government declared a general holiday instead of a lockdown at the beginning [34]. The term ‘general holiday’ prevents people to understand the importance of effective lockdown, that’s why people felt free to roam around and ignored social distancing guidelines. This might be the cause of poor practice scores because social controlling measures were found as a significantly associated factor with the practice of preventive measures [24].

In addition to other factors, this study also identified that good knowledge and positive attitude were significantly associated with good practice. Globally many other studies also agreed with this statement [11, 21, 24, 42]; which suggested that health education can play a vital role in promoting preventive practice toward COVID-19 [24, 34]. Moreover, this study also found that women are more likely to have preventive practices other than men and the lower age group’s respondents showed good practice scores compared with older, which was consistent with a study in Iran [25]. Alternatively, regression analysis showed that male older adults, who completed only primary education, aged above 70 years, had lower family income, lived in a rural area, had multimorbidity, poor knowledge of COVID-19, and negative attitudes were more likely to have poor practice; as similar to other previous studies [26, 39].

Considering other studies, it has been seen that older people are less informed and adopted prevention practices for COVID-19 [26, 34]. Moreover, aged people are more vulnerable to severe diseases condition and death from COVID-19 [43]. Bangladesh is a multi-ethnic country and respondents come across different socioeconomic levels such as education, income, family size, and different living area, which markedly influenced the knowledge, attitude, and practice of older adults of Bangladesh. Although we found a good knowledge and attitude score, the practice score was not at a satisfactory level. As because practices were associated with knowledge and attitude, so we recommended that an easy and older age-friendly social behavior change communication (SBCC) strategy should be developed. Additionally, novel information should have to be available on every social communication platform like the internet, radio, television, and newspaper. It is very important to disseminate accurate information otherwise it will negatively affect the practices and make people confused. To improve KAP among elderly people in Bangladesh a comprehensive national action plan should be developed which may include a specific preventive message, way of message delivery, community-level service delivery team, and strategic plan to reach the rural area as well. The government has to consider elderly people as a highly vulnerable group during strategic planning and take action accordingly.

### Strength and limitations

The study’s strength is its large sample size, which was obtained during a critical period of nationwide lockdown and the COVID-19 outbreak. Data were collected from five administrative divisions across the country, and participants were surveyed in person in both rural and urban areas, which was a major strength of our study. The findings were more generalizable to the Bangladeshi older adults as a result of this data collection process.

There are some limitations to this study that should be considered. This study used a cross-sectional design, which cannot establish causal inferences. As a result, a longitudinal study would be able to overcome this barrier to understanding possible causal relationships. Responses (primarily attitudes and practices) could have been reported based on social desirability, not the actual situation of the participants, as in any other self-reported study. Despite these limitations, the study’s findings are expected to encourage and immediately notify policymakers and program implementers who are working on appropriate risk communication and community engagement based on KAP levels for COVID-19.

## Conclusion

In summary, the present research revealed the level of knowledge, attitudes, and practices among elderly Bangladeshi people and also identified possible socioeconomic indicators that may be associated with their KAP. Our findings showed that a considerable number of older participants in Bangladesh were adequately knowledgeable regarding COVID-19 and had a positive attitude, but surprisingly poor practices measures, which can be alarming and very important factors for limiting the spread of diseases like COVID-19. Furthermore, there was a significant association among male, oldest people, low-income, rural residents, and employed respondents significantly associated with poor knowledge and practices, which highlights the need for an effective, age-friendly, easy-to-understand health education program among healthcare workers and policymakers for improving knowledge on COVID-19 preventive measures for aged people. Generally, appropriate knowledge leads to a satisfactory attitude and maintaining safe practices. So, the evidence advised a strategic support system for older people may reduce the consequences of health emergencies like COVID-19.
